# Discontinuation of Azathioprine could be considered in pediatric patients with Crohn’s disease who have sustained clinical and deep remission

**DOI:** 10.1038/s41598-021-04304-6

**Published:** 2022-01-11

**Authors:** Tae Jong Jeong, Eun Sil Kim, Yiyoung Kwon, Seonwoo Kim, Sang Won Seo, Yon Ho Choe, Mi Jin Kim

**Affiliations:** 1 Department of Pediatrics, Samsung Medical Center, Sungkyunkwan University School of Medicine, Seoul, Korea; 2 Statistics and Data Center, Samsung Medical Center, Seoul, Korea; 3 Department of Neurology, Samsung Medical Center, Sungkyunkwan University School of Medicine, Seoul, Korea; 4 Neuroscience Center, Samsung Medical Center, Seoul, Korea; 5 Department of Clinical Research Design and Evaluation, SAIHST, Sungkyunkwan University, Seoul, Korea

**Keywords:** Diseases, Gastroenterology, Risk factors

## Abstract

Few studies have demonstrated treatment strategies about the duration and cessation of medications in patients with Crohn’s disease (CD). We investigated factors affecting clinical relapse after infliximab (IFX) or azathioprine (AZA) withdrawal in pediatric patients with CD on combination therapy. Pediatric patients with moderate-to-severe CD receiving combination therapy were analyzed retrospectively and factors associated with clinical relapse were investigated. Discontinuation of IFX or AZA was performed in patients who sustained clinical remission (CR) for at least two years and achieved deep remission. A total of 75 patients were included. Forty-four patients (58.7%) continued with combination therapy and 31 patients (41.3%) discontinued AZA or IFX (AZA withdrawal 10, IFX withdrawal 15, both withdrawal 6). Cox proportional-hazards regression and statistical internal validation identified three factors associated with clinical relapse: IFX cessation (hazard ratio; HR 2.982, *P* = 0.0081), IFX TLs during maintenance therapy (HR 0.581, *P* = 0.003), 6-thioguanine nucleotide (6-TGN) level (HR 0.978, *P* < 0.001). However, AZA cessation was not associated with clinical relapse (*P* = 0.9021). Even when applied in pediatric patients who met stringent criteria, IFX cessation increased the relapse risk. However, withdrawal of AZA could be contemplated in pediatric patients with CD who have sustained CR for at least 2 years and achieved deep remission.

## Introduction

Crohn’s disease (CD) is a chronic, relapsing disease of the gastrointestinal tract that can cause serious morbidity and disability^1^. Current treatment trends recommend the early introduction of biologics in pediatric patients with CD exhibiting severe disease^2^. Infliximab (IFX), one type of anti-tumor necrosis factor (TNF) agent, can be appropriate initial therapy in moderate-to-severe pediatric CD patients^3^. It is well known that corticosteroid-free clinical remission (CR) is more likely to be achieved in patients with moderate-to-severe CD when treated with a combination therapy that includes IFX plus azathioprine (AZA) or with IFX monotherapy, than in those receiving AZA monotherapy^4^. Early introduction of IFX, known as top-down therapy, is an important option for pediatric patients with moderate-to-severe CD and has become the mainstay of treatment for CD^5,6^. However, since long-term use of AZA increases the risk of complications such as leukopenia, opportunistic infections, and lymphoma in children, concomitant use of immunomodulators and biologics has raised safety concerns^7,8^. In addition, combination therapy is not clearly associated with high efficacy after achieving long-term remission^9^. Despite the increasing use of combination therapy, few studies have established the optimum strategy in terms of duration and cessation of medications after achieving remission, which is still a matter of discussion^10^. Furthermore, although it is well known that concomitant treatment with immunomodulator reduces the formation of antibody-to-infliximab (ATI) and improves the pharmacokinetics of IFX, other factors affecting ATI formation are not clearly identified^11^.

The aim of this study was to determine factors affecting clinical relapse after achieving remission in pediatric CD and to estimate the proper duration and time of cessation of early combination therapy in a selected population of pediatric patients with CD in a real-life cohort.

## Materials and methods

### Study design and patients

This was a retrospective observational study conducted at the Department of Pediatrics of Samsung Medical Center in the Republic of Korea, between January 2012 and March 2018. Eligible patients had moderate-to-severe luminal CD and were aged less than 19 years at both time of diagnosis and the time of their first treatment with IFX. CD was diagnosed in accordance with the revised Porto criteria of the European Society for Pediatric Gastroenterology, Hepatology and Nutrition^12^ and disease phenotype classification was based on the Paris classification^13^. Patients on early combination therapy were included in the analysis, and patients underwent endoscopy on a regular basis every 1–2 years.

Patients were excluded if they were primary non-responders to IFX, or developed ATI during induction therapy of IFX. In addition, patients who were poor metabolizers of AZA according to thiopurine methyltransferase (TPMT) and nucleotide triphosphate diphosphatase 15 (NUDT15) genotype, were excluded for accurate analysis of effects of combination therapy^14^.

IFX was administered according to the scheduled induction regimen of 5 mg/kg at weeks 0, 2, and 6, and scheduled maintenance IFX was repeated every 8 weeks. Twenty-six patients were treated with originator Remicade®, 35 patients treated with Remsima®, a biosimilar version of IFX, and 14 patients switched from Remicade to Remsima during the follow up period. Since there was no difference in the clinical efficacy of Remicade and Remsima according to the previous studies^15–17^, it was considered that there was no difference according to the type of administered IFX. IFX doses were adjusted by IFX TL during maintenance therapy; if IFX TL was less than 3 μg/ml, dose intensification was performed. Azathioprine was given at doses of 0.5–1 mg/kg/day and was later modified when required according to TPMT and NUDT15 genotype and thiopurine metabolite levels of 6-thioguanine nucleotide (6-TGN) levels (therapeutic range: 235–450)^18^.

Drug cessation was performed among patients who maintained CR more than two years and at the same time achieved deep remission. The decision to discontinue either IFX or AZA was determined by various requirements, such as concerns about side effects from long-term use of the medication, cost effectiveness, and patients’ and parental demands. We did not selectively discontinue specific medications for specific patients.

### Data collection and definitions

Demographic and clinical data at diagnosis, at combination therapy initiation, at drug cessation, and during follow-up were collected from electronic medical records: age, sex, disease phenotype, body mass index (BMI), pediatric Crohn’s disease activity index (PCDAI), laboratory results, simple endoscopic score for CD (SES-CD), histologic results and concomitant medication. Date related to AZA or IFX cessation, date of clinical relapse, 6-TGN, IFX TLs, and formation of ATI were collected retrospectively from electronic charts or electronic test results. The data described above was used to determine factors affecting clinical relapse and to estimate the appropriate duration and time of cessation of early combination therapy.

Serum samples were obtained prior to each infusion for measurement of IFX TLs and ATI. IFX TLs and ATI had been quantified using an enzyme-linked immunosorbent assay (ELISA; Matriks Biotek Laboratories, Ankara, Turkey). We used sandwich ELISA to measuring IFX TLs and free ATI. We measured free ATI which is known to be associated with low serum IFX TLs and probability of active disease using drug-sensitive ATI assays^19^. AZA concentration was estimated indirectly every 3–6 months by measuring 6-TGN levels which are considered to be the primary active metabolite of AZA.

CR was defined as a PCDAI < 10 points. Deep remission was defined as the absence of mucosal ulceration (SES-CD < 2), i.e., mucosal healing, at the same time as the achievement of histologic remission which means the absence of microscopically active inflammation in all gastrointestinal tissue obtained by endoscopy. Clinical relapse was defined as a PCDAI score of ≥ 10 with a change of at least 10 points from the previous visit with the need for treatment intensification, defined as either i) the addition of a new medication, (ii) dose escalation of maintenance treatment and (iii) intestinal surgery because of stricturing or penetrating CD.

### Statistical analysis

Baseline characteristics of subjects were explored with descriptive statistics through frequencies (proportion) for categorical variables, or medians (interquartile range; IQR) for continuous variables. Data related to clinical characteristics, the duration and cessation timing of medication, 6-TGN, and IFX TLs were analyzed. Data with normal distribution and/or equal variances (Levene’s and F-test for equality of variances) were analyzed with 1-way ANOVA or *t*-test for independent samples, whereas data without normal distribution were analyzed with Kruskal–Wallis *H*-test or Mann–Whitney *U*-test. The risk factors associated with clinical relapse were identified by Cox proportional-hazards regression models and internally validated by bootstrap resampling^20^. Time-varying covariance occurs when covariates change over time during the follow-up period. Drug-related factors were more appropriate to be analyzed as time-varying covariates rather than time-independent covariates because variables such as drug administration (or discontinuation) and drug concentration vary over time. Therefore, cessation and duration of medication, 6-TGN, and IFX TLs were analyzed using time-varying covariates with the Cox regression model to estimate these effects on survival time. For this, we organized the data in a counting process style. Hazard ratio (HR) for each variable was derived within 95% confidence intervals (CIs). Relapse-free curve was estimated using an extended Kaplan–Meier method that can be used with time-varying covariates^21^. Data were considered significantly different if two-sided *P* was < 0.05. All statistical analyses were performed using SAS software (version 9.4; SAS Institute Inc., Cary, NC, USA) and R software (version 3.6.1; R Foundation, Vienna, Austria).

### Ethics declarations

This study was approved by the Institutional Review Board of the Samsung Medical Center (IRB file No. 2020–06-128–001), and was conducted in accordance with the Declaration of Helsinki. All patients and parents and/or legal guardian of subjects who are under 18 provided written informed consent. We confirmed that all methods were performed in accordance with the approved guidelines and regulations. We reported and presented data according to the STROBE statement.

## Results

### Baseline characteristics

**Figure 1:**
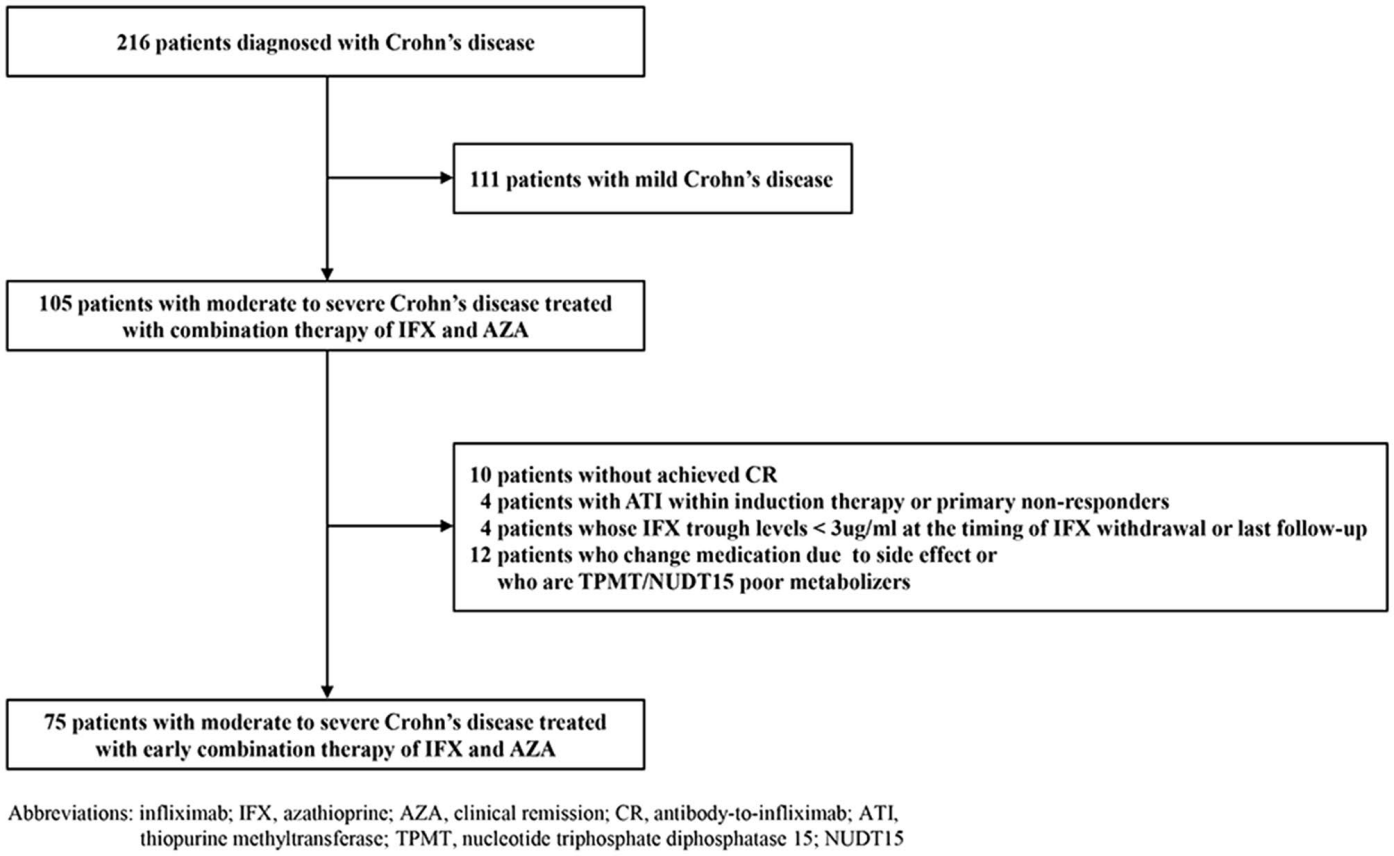
Flow diagram showing patient selection process. AZA, azathioprine; IFX, infliximab; ATI, antibody-to-infliximab.

From January 2012 to March 2018, a total of 216 pediatric patients were diagnosed with CD and of these 75 patients were finally considered eligible for analysis as shown in the flow diagram for patient selection (Fig. 1). Among the study participants, 48 patients (64.0%) were male and the median age of subjects at diagnosis was 14.2 years (IQR 12.0–17.0). The median initial PCDAI at diagnosis was 39.7 (IQR 37.5–45.0) and median initial SES-CD was 16.9 (IQR 11.0–24.0). The median observational duration was 41.5 months (IQR 23.0–58.7 months). Other baseline characteristics are described in detail in Table 1.

**Table 1:** Baseline clinical characteristics of study patients.

	Total (*n* = 75)
**Gender,** ***n *** **(%)**	
Male Female	48 (64.0) 27 (36.0)
**Observational period, *months***	41.5 (23.0, 58.7)
**Age at diagnosis, *years***	14.2 (12.0, 17.0)
**Age group, *n* (%)**	
5–9 years 10–14 years 15–19 years	5 (6.6) 35 (46.7) 35 (46.7)
**Initial BMI at diagnosis, kg/m**^**2**^	19.0 (16.8, 20.7)
**Initial PCDAI at diagnosis**	39.7 (37.5, 45.0)
**Disease location, *n* (%)**	
Ileal (L1) Colonic (L2) Ileocolonic (L3)	9 (12.0) 3 (4.0) 61 (81.3)
**Upper gastrointestinal involvement, *n* (%)**	
None Proximal to the ligament of Treitz (L4a) Distal to the ligament of Treitz and proximal to the distal 1/3 ileum (L4b) Both (L4ab)	1 (1.3) 9 (12.0) 9 (12.0) 56 (74.7)
**Behavior of disease, *n* (%)**	
Inflammatory (B1) Stricturing (B2) Penetrating (B3)	52 (69.3) 20 (26.7) 3 (4.0)
**Growth delay,* n *(%)**	
No evidence of growth delay (G0) Growth delay (G1)	51 (68.0) 24 (32.0)
**Perianal disease, *n* (%)**	43 (57.3)
**Initial Laboratory findings**	
White blood cell count, × 10^3^/μL Hematocrit, % Platelet count, × 10^3^/μL Erythrocyte sedimentation rate, mm/h C-reactive protein, mg/dL Albumin, g/dL	8.8 (6.7, 11.1) 36.8 (33.4, 39.8) 382 (309, 491) 55.0 (29.5, 77.5) 3.1 (0.8, 4.3) 3.8 (3.4, 4.3)
**Initial SES-CD at diagnosis**	16.9 (11.0, 24.0)
**Concomitant medication, *n* (%)**	
Mesalazine	73 (97.3)

Baseline characteristics of subjects were explored with descriptive statistics through frequencies (proportion) for categorical variables or medians (interquartile range[IQR]) for continuous variables.

BMI, body mass index; PCDAI, pediatric Crohn’s disease activity index; SES-CD, simple endoscopic score for Crohn’s disease; 6-TGN, 6-thioguanine nucleotide.

### Relapse rate of patients according to withdrawal of medications

Of 75 patients, 31 (41.3%) patients met the criteria of sustained CR more than two years and the definition of deep remission, and discontinued AZA or IFX according to various requirements. Sixteen patients withdrew AZA, 21 patients IFX, and among them, six patients discontinued both. The remaining 44 patients (58.7%) who achieved CR but did not reach deep remission, continued combination therapy with IFX and AZA. The mean durations of AZA and IFX therapy were 38.0 ± 19.3 months and 32.0 ± 18.9 months respectively. In the drug discontinuation group, the mean follow-up duration after AZA and IFX withdrawal was 14.0 ± 9.5 months and 28.0 ± 22.9 months respectively. When comparing the group that withdrew AZA with the group who discontinued IFX, there was no significant difference in disease activity and laboratory results at the time of diagnosis and at the time of drug discontinuation (Table 2).

**Table 2:** Comparison between patients discontinuing infliximab or azathioprine.

	IFX withdrawal group (*n* = 15)	AZA withdrawal group (*n* = 10)	IFX and AZA withdrawal group (*n* = 6)	*P*-value
Sex, male (%)	7 (31.80)	9 (40.90)	6 (27.30)	0.018^Chi^
**At diagnosis**				
White blood cell count, × 10^3^/μL	7.80 (7.43, 9.71)	9.29 (8.23, 10.72)	8.61 (7.80, 9.19)	0.673^ K^
Hematocrit, %	35.60 (33.10, 39.10)	35.20 (32.30, 39.10)	40.00 (37.90, 41.70)	0.069 K
ESR, mm/h	53.00 (41.50, 78.0)	63.00 (39.30, 83.50)	25.00 (23.00, 29.30)	0.071 K
C-reactive protein, mg/dL	1.21 (0.64, 7.60)	2.37 (1.84, 3.52)	2.33 (1.10, 3.46)	0.712 K
Albumin, g/dL	3.80 (3.60, 4.30)	3.80 (3.50, 4.30)	4.20 (4.00, 4.40)	0.479 K
PCDAI at diagnosis	40.00 (33.30, 46.30)	35.00 (30.60, 41.90)	35.00 (30.00, 41.00)	0.515 K
SES-CD at diagnosis	16.00 (11.50, 19.50)	18.00 (14.30, 24.00)	13.50 (7.00, 17.80)	0.298 K
**At IFX or AZA withdrawal**				
White blood cell count, × 10^3^/μL	5.52 (4.80, 7.07)	6.11 (5.45, 6.88)	5.67 (5.11, 5.82)	0.830 K
Hematocrit, %	39.40 (37.10, 43.80)	45.40 (44.50, 46.60)	43.20 (42.50, 45.50)	0.174 K
ESR, mm/h	12.00 (4.50, 20.50)	4.50 (2.30, 9.00)	5.00 (2.80, 11.80)	0.243 K
C-reactive protein, mg/dL	0.03 (0.03, 0.08)	0.04 (0.03, 0.04)	0.13 (0.03, 0.36)	0.437 K
Albumin, g/dL	4.50 (4.40, 4.70)	4.70 (4.60, 4.90)	4.50 (4.50, 4.60)	0.196 K
PCDAI at drug withdrawal	0 (0, 0)	0 (0, 0)	0 (0, 0)	0.669 K
SES-CD at drug withdrawal	0 (0, 0.45)	0 (0, 0)	0 (0, 0.25)	0.496 K

Baseline characteristics of subjects who withdrew infliximab or azathioprine were explored with descriptive statistics through frequencies (proportion) for categorical variables or medians (interquartile range[IQR]) for continuous variables.

^Chi^χ^2^ test; ^K^ Kruskal–Wallis test.

ESR, Erythrocyte sedimentation rate; PCDAI, Pediatric Crohn’s disease activity index; SES-CD, Simple endoscopic score for Crohn’s disease; AZA, azathioprine; IFX, infliximab.

Among 75 patients, 31 (41.3%) were clinically relapsed and 44 patients (58.7%) maintained CR during the study period. Four of 16 patients who withdrew AZA experienced clinical relapse (4/16, 25.0%), 15 of 21 patients who withdrew IFX experienced relapse (15/21, 71.4%), and four of six patients who withdrew all drugs experienced relapse (4/6, 66.6%). Sixteen of 44 patients (16/44, 36.4%) who continued IFX and AZA during study period experienced relapse.

### Factors associated with clinical relapse

A Cox proportional-hazards regression identified four factors associated with clinical relapse. IFX cessation (HR 2.982, 95% CI 1.322–6.485, *P* = 0.0081), formation of ATI (HR 3.120, 95% CI 1.069–9.103, *P* = 0.0373), low IFX TLs during maintenance therapy (HR 0.581, 95% CI 0.432–0.781, *P* = 0.0003) and low 6-TGN levels (HR 0.978, 95% CI 0.968–0.987, *P* < 0.0001) were found to increase the risk of clinical relapse. AZA cessation (HR 1.078, 95% CI 0.327–3.550, *P* = 0.9021) was not shown to increase the risk of clinical relapse (Table 3). In the analysis using bootstrapped data for internal validation, IFX cessation (HR 3.178, 95% CI 1.294–6.312), low 6-TGN level (HR 0.941, 95% CI 0.267–0.988), low IFX TLs (HR 0.544, 95% CI 0.031–0.765) were confirmed to be associated with clinical relapse, whereas ATI formation was not shown to increase risk of clinical relapse in bootsrapped data (HR 5.869, 95% CI 0.775–22.741).

**Table 3:** Factors affecting clinical relapse in pediatric patients with Crohn’s disease.

Variables	Univariate Cox analysis			Bootstrapped data (*n* = 500)	
	HR	95% CI	*P*-value	HR	95% CI
Age	0.965	0.854–1.091	0.5666	0.967	0.845–1.108
**Sex**					
Male	Reference			Reference	
Female	1.203	0.579–2.500	0.6204	1.287	0.579–2.488
**Initial laboratory findings**					
White blood cell counts	0.676	0.206–2.218	0.5184	0.897	0.192–3.150
Hematocrit	0.960	0.890–1.035	0.2872	0.962	0.889–1.042
Albumin	0.751	0.408–1.381	0.3574	0.779	0.387–1.304
Erythrocyte sedimentation rate	1.009	0.998–1.020	0.1234	1.009	0.999–1.018
C-reactive protein	0.964	0.865–1.074	0.5056	0.969	0.898–1.092
Initial PCDAI	1.004	0.971–1.037	0.8282	1.005	0.973–1.044
Initial SES-CD	0.995	0.954–1.038	0.8209	0.997	0.954–1.049
Initial disease phenotype					
L1 (Ileal) + L3 (Ileaocolonic)	Reference			Reference	
L2 (Colonic)	1.996	0.472–8.440	0.3472	5.190	0.277–23.038
**Initial disease behavior**					
B2 (Stricturing) + B3 (Penetrating)	Reference			Reference	
B1 (Inflammatory)	0.891	0.408–1.948	0.7727	0.951	0.390–1.966
Initial growth impairment	0.669	0.293–1.483	0.3139	0.722	0.257–1.475
*AZA cessation	1.078	0.327–3.550	0.9021	1.264	0.312–3.086
*IFX cessation	2.928	1.322–6.485	0.0081	3.178	1.294–6.312
*Mesalazine cessation	1.165	0.517–2.627	0.7129	1.334	0.504–3.054
*antibody-to-IFX formation	3.120	1.069–9.103	0.0373	5.869	0.775–22.741
*IFX Trough levels	0.581	0.432–0.781	0.0003	0.544	0.031–0.765
*6-TGN levels	0.978	0.968–0.987	< 0.0001	0.941	0.267–0.988

The risk factors associated with clinical relapse were identified by Cox proportional-hazards regression models. In this model, cessation and duration of medication, 6-TGN, and IFX TL were analyzed as time-varying covariates. Hazard ratio (HR) for each variable was derived within the 95% confidence intervals (CIs).

*Variables were analyzed as time varying covariates.

PCDAI, pediatric Crohn’s disease activity index; SES-CD, simple endoscopic score for Crohn’s disease; AZA, azathioprine; IFX, infliximab; 6-TGN, 6-thioguanine nucleotide

**Figure 2:**
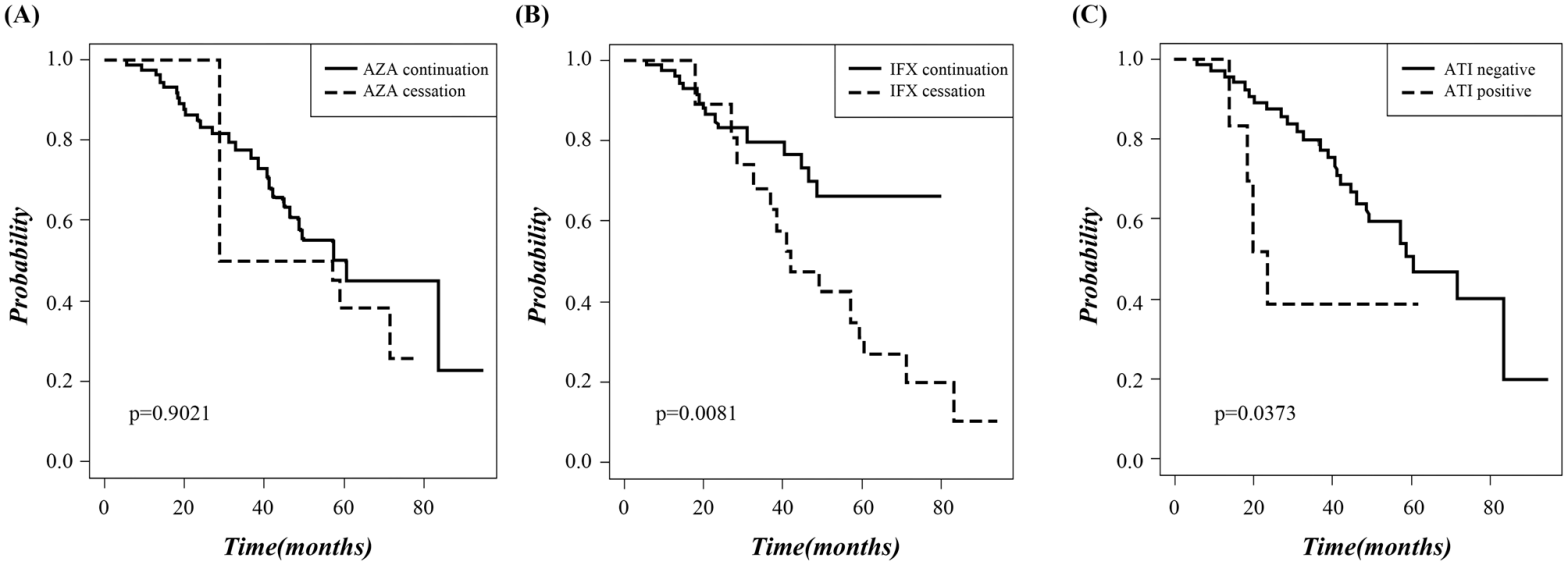
Relapse-free curve according to drug cessation and development of antibody-to-infliximab. AZA, azathioprine; IFX, infliximab; ATI, antibody-to-infliximab.

To evaluate the relapse-free curve in relation to discontinuation of medication and ATI formation, Kaplan–Meier survival curves were calculated (Fig. 2). Patients who discontinued IFX (Fig. 2B, *P* = 0.0081) or developed ATI (Fig. 2C, *P* = 0.0373) had significantly poorer outcomes compared to those who did not. However, there was no difference in survival rate between patients who continued or discontinued AZA (Fig. 2A, *P* = 0.9021).

### Factors associated with formation of antibody-to-infliximab

**Figure 3:**
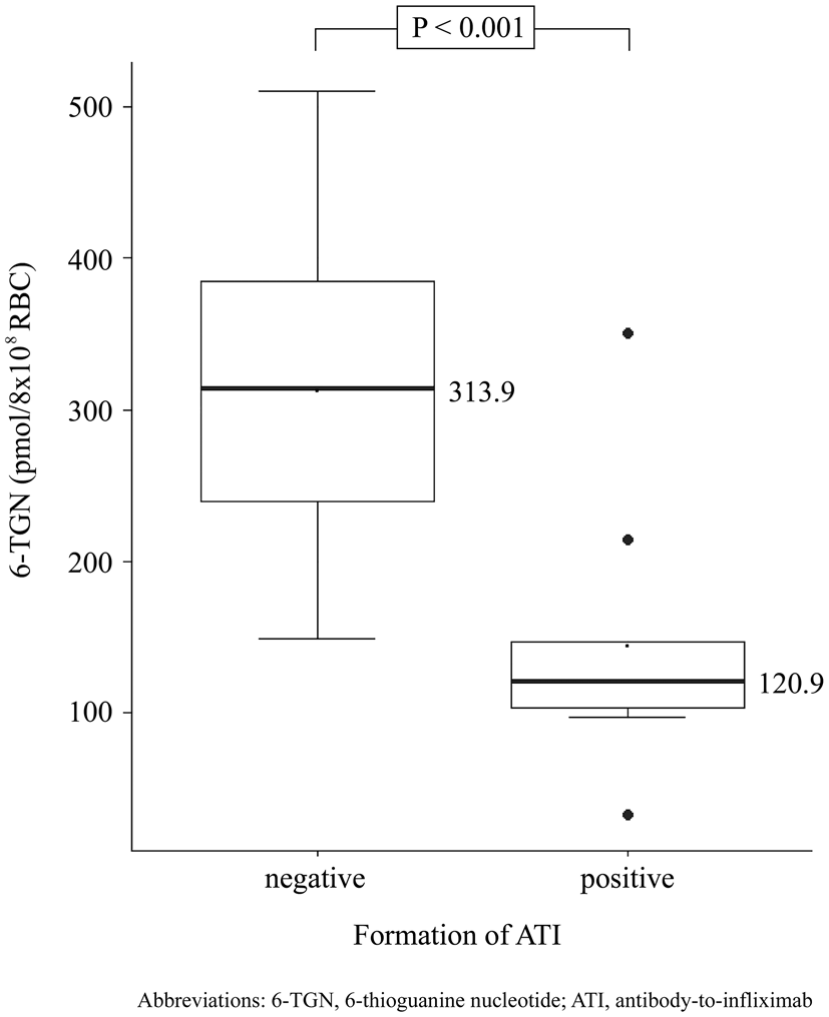
X6-Thioguanine nucleotide levels according to formation of antibody-to-infliximab. 6-TGN, 6-thioguanine nucleotide; ATI, antibody-to-infliximab.

The formation of ATI was detected in nine patients (12.0%) receiving combination therapy. Factors associated with formation of ATI during maintenance therapy were evaluated. According to Cox proportional-hazards regression, only 6-TGN level was related to the formation of ATI (HR 0.988, 95% CI 0.980–0.996, *P* = 0.0048). AZA cessation (HR 17.99, 95% CI 0.194–1663.717, *P* = 0.2109) was not related to formation of ATI. Within the therapeutic range (235–450 pmol/8 × 10^8^ RBC)^18^, high 6-TGN level is a factor that lowers the risk of developing ATI (HR 0.988, 95% CI 0.980–0.996, *P* = 0.0048, Table 4). In addition, analysis using bootstrap for internal validation also found that low 6-TGN is associated with risk of ATI formation (HR 0.978, 95% CI 0.929–0.998). When comparing patients according to the formation of ATI, median 6-TGN level is higher in ATI negative group than ATI positive group (313.9 vs. 120.9, *P* < 0.001, Fig. 3).

**Table 4:** Factors affecting development of antibody-to-infliximab.

Variables	Univariate Cox analysis			Bootstrapped data (*n* = 500)	
	**HR**	**95% CI**	***P*-value**	**HR**	**95% CI**
Age	1.132	0.872–1.468	0.3511	1.169	0.852–1.696
**Sex**					
Male	Reference			Reference	
Female	1.561	0.419–5.813	0.5072	2.116	0.381–6.628
**Initial laboratory findings**					
White blood cell counts	0.456	0.041–5.108	0.5239	1.001	0.044–4.311
Hematocrit	1.033	0.900–1.185	0.6460	1.048	0.911–1.237
Albumin	0.859	0.303–2.436	0.7751	0.924	0.401–1.960
Erythrocyte sedimentation rate	1.004	0.983–1.025	0.7192	1.004	0.986–1.024
C-reactive protein	0.992	0.812–1.211	0.9340	0.981	0.720–1.178
Initial PCDAI	1.001	0.943–1.063	0.9658	1.001	0.959–1.040
Initial SES-CD	0.950	0.875–1.030	0.2150	0.940	0.816–1.041
**Initial disease phenotype**					
L1 (Ileal) + L3 (Ileaocolonic)	Reference			Reference	
L2 (Colonic)	3.297	0.410–26.513	0.2620	6.503	0.874–24.412
**Initial disease behavior**					
B2 (Strictureing) + B3 (Penetrating)	Reference			Reference	
B1 (Inflammatory)	0.661	0.137–3.183	0.6058	0.924	0.126–2.800
Initial growth impairment	1.115	0.279–4.462	0.8775	1.508	0.159–4.441
*AZA cessation	17.987	0.194–1663.717	0.2109	11.249	2.890–19.997
*6-TGN level	0.988	0.980–0.996	0.0048	0.978	0.929–0.998

The risk factors associated with formation of ATI were identified by Cox proportional-hazards regression models. In this model, cessation of medication and 6-TGN were analyzed as time-varying covariates. Hazard ratio (HR) for each variable was derived within the 95% confidence intervals (CIs).

*Variables were analyzed as time varying covariates.

PCDAI, pediatric Crohn’s disease activity index; SES-CD, simple endoscopic score for Crohn’s disease; AZA, azathioprine; 6-TGN, 6-thioguanine nucleotide.

## Discussion

We investigated the factors affecting clinical relapse and estimated the optimum duration and time of cessation of medication in moderate to severe pediatric CD treated with combination therapy. We identified three factors related to elevated risk of clinical relapse; IFX cessation, low IFX TLs, low 6-TGN level. Interestingly, withdrawal of AZA was not directly associated with clinical relapse in pediatric CD patients with sustained CR of at least two years and who had achieved deep remission.

In clinical practice, physicians may encounter questions about the feasibility of therapeutic de-escalation in CD patients who have achieved deep remission, on account of safety, adverse events and cost. There is a paucity of data related to the optimum duration and the appropriate time to cease medication after achieving remission in patients with CD, particularly in pediatric patients.

It is now known that early introduction of biologics has tremendous advantages in terms of maintaining tight control of inflammation and catching the therapeutic window^22,23^. A randomized controlled trial (RCT) revealed that co-treatment with IFX and AZA significantly increases the rate of CR and mucosal healing compared to AZA monotherapy in CD patients^4^. In addition, recent studies have reported that endoscopic and histological mucosal healing should be treat-to-target in CD patients instead of symptomatic control to achieve better outcomes and minimize future complications^24^. With the development of treatment strategies, clinicians are being confronted with a crossroads decision about when to stop or reduce the therapy dose once patients achieve remission^25^.

A systematic review of cessation studies for patients with inflammatory bowel disease (IBD) concluded that about 50 percent of patients who withdrew anti-TNF-α agents after combination therapy with immunomodulators maintained remission for 24 months^26^. Reenaers et al*.* reported that among patients with CD who withdrew IFX in stable CR state, twenty-one percent did not restart biologics including IFX, and sustained CR for seven years after IFX cessation^27^. Therefore, it seems reasonable to conclude that there may be a subgroup of patients who are good candidates for treatment withdrawal.

Our data that IFX cessation in patients with CD was associated with a high risk of clinical relapse is consistent with the results of other previously published studies^28–31^. A recent retrospective study conducted in Korea on adults evaluated the long-term outcomes following cessation of anti-TNF-α treatment in IBD patients with CR^30^. After cessation of anti-TNF-α treatment for CD patients, the cumulative relapse rates at 1, 3, and 5 years were 11.3%, 46.7%, and 62.5%. In this cohorts, mucosal healing rate before discontinuation of anti-TNF-α treatment in CD patents was 22.5%. Another recent retrospective study performed by Luca and colleagues investigated the clinical course after anti-TNF-α treatment discontinuation in selected pediatric patients with IBD who achieved deep remission similar to our study^31^. Among these patients, relapse-free survival rates at 12, 24, and 36 months for CD were 83.3%, 71.1%, and 23.7%, respectively. In contrast, none of the patients with CD who maintained anti-TNF-α treatment after achieving mucosal healing experienced clinical relapse^30^.

However, the association between discontinuation of AZA and clinical relapse is controversial. In one reported study, which was focused on CD in CR under IFX-AZA combination therapy, AZA cessation was associated with a high risk of relapse in those patients treated with combination therapy for less than 27 months^32^. In contrast, two other RCTs and one meta-analysis suggested that discontinuing AZA from a combination therapy regimen may not differ in clinical relapse rates compared to continuing with combination therapy^9,26,33^. However, both RCTs included subjects comprised primarily of those who had previously failed immunomodulators monotherapy. It could be argued that in these cohorts, the subsequent cessation of this agent would not be expected to have a significant effect compared to a patient cohort in which patients were initially treated with combination therapy^10^. Nevertheless, most studies of patients with CD who discontinued AZA after combination therapy revealed that there were no differences between AZA withdrawal from combination therapy and continuation of combination therapy in terms of clinical relapse. In addition, the European Crohn’s and colitis organization guideline provide the opinion that AZA withdrawal in patients treated with combination therapy is inappropriate in patients with high risk/refractory disease or in patients at risk of biologic failure^34^. In other words, it can be said that in patients with sustained deep remission, controlled disease activity and low risk of biologic failure, azathioprine could be withdrawn.

When subjects were treated with combination therapy and maintained CR for at least two years, mucosal healing was observed in 82.7% and deep remission was achieved in 41.3% in our study. Based on these findings, we attempted drug cessation assuming that most of the patients who satisfied both conditions had received sufficient treatment and had a low risk of relapse. In our study, AZA cessation was not shown to increase the risk of clinical relapse (HR 1.078, 95% CI 0.327–3.55, *P* = 0.9021). This result could be partially explained by another study which revealed that withdrawal of AZA after at least six months of combination therapy does not reduce IFX TLs in patients with CD^35^. The mean durations of AZA therapy were 38 ± 19.3 months. We also wanted to know the change in the risk of relapse according to the treatment duration with AZA, but the number of patients who discontinued AZA was small, so further statistical analysis was impossible.

According to our results and those of other reports, IFX TLs and 6-TGN levels affect clinical relapse^36,37^. High drug concentrations within the therapeutic range could be a factor in lowering the risk of relapse. Especially, it has been reported that IFX TLs are positively associated with mucosal healing and could be a factor in lowering the risk of relapse during maintenance treatment with IFX^38^. Pursuing a high therapeutic concentration may lead to concerns about adverse drug reactions. However, using therapeutic drug monitoring (TDM) for personalizing therapy for CD patients, drug concentration could be maintained within the therapeutic range. The average IFX TLs and 6-TGN levels during the follow-up period were 6.1 ± 5.4 μg/mL and 208.5 ± 114.2 pmol/8 × 10^8^ RBC respectively. Since patients who were poor metabolizers of AZA and primary non-responders to IFX were excluded from this study, the relationship between higher drug concentrations above therapeutic range of 6-TGN and occurrence of adverse events was not addressed in this study. Interestingly, in our study, IFX TLs at the time of IFX cessation were significantly higher in patients who experienced relapse than patients who maintained CR (5.4 ± 2.8 vs. 2.8 ± 0.8, *P* < 0.01). The present findings correlate with the findings of previous studies, which indicate higher IFX TLs at IFX cessation were associated with relapse^39,40^.

In adults, the development of ATI occurs in up to 65.3% in patients with IBD^41^, while ATI have been reported in 8% to 43% of pediatric patients with IBD^42^. The results of our study are consistent with these data, as the formation of ATI was detected in 12.0% of patients receiving combination therapy. The development of ATI can neutralize IFX by direct binding of neutralizing antibodies, or accelerating the clearance of the IFX by the binding of non-neutralizing antibodies^43,44^. These mechanisms lower IFX TLs and can lead to loss of response during IFX administration^45^. Similarly, patients with ATI formation had a lower relapse free survival rate than those with negative ATI in our study (Fig. 3C). ATI formation was shown to increase risk of clinical relapse in univariate Cox analysis in our data, whereas it was not shown to increase risk of clinical relapse in bootstrapped data. Since the number of ATI positive patient is small (*n* = 9), the estimation and results are considered unreliable. Therefore, it is thought that confirmation through a study using a larger cohort is necessary in the future.

It is well known that concomitant use of immunomodulators reduces ATI formation, and improves the pharmacokinetics of IFX^9,46,47^. This finding is interesting because it appears to be related with the role of AZA in preventing immunogenicity. Recent cross-sectional study of 72 patients receiving maintenance therapy with IFX and a thiopurine for IBD concluded that patients with 6-TGN levels less than 125 pmol/8 × 10^8^ RBC were significantly more likely to have ATI (odds ratio 1.3, 95% CI 2.3–72.5; *P* < 0.01)^48^. 6-TGN levels of 125 pmol/8 × 10^8^ RBC or higher were best predictive of the absence of ATIs; higher 6-TGN concentrations did not provide additional benefit at least in terms of ATI formation. However, other factors affecting ATI formation were not clearly identified. We specifically analyzed whether there is a relationship between the factors affecting clinical relapse and ATI formation. According to our analysis, only 6-TGN level was associated with an increased risk of formation of ATI and this result is consistent with another study^49^.

In our study, AZA cessation (HR 17.99, P = 0.2109) was not associated with formation of ATI. However, with a hazard ratio of 17.99, this affirmation does not seem reliable, in spite of P > 0.05 because of small population. In addition, from the results estimated from the bootstrapped data, AZA cessation was found to increase the risk of ATI development. Considering the role of AZA in preventing immunogenicity, AZA cessation might be a risk for ATI formation theoretically. It is thought that this is because the number of ATI-positive patients in outcome and the number of patients who discontinued AZA were small, and the estimation and results could be unreliable.

Although the present study is retrospective and relatively small-sized, it sought a more analytical approach to drug cessation and drug concentration. Drug-related factors were analyzed as time-varying covariates rather than time-independent covariates. Conventional survival analysis is generally applied to the time-independent data, where the exposure variables of interest are often treated as time-fixed^50^. However, values of these exposure variables can vary over time and time-fixed analysis may cause bias over time potentially altering the conclusions of the study. To the best of our knowledge, none of the studies to determine when to withdraw drugs in patients with CD have used time-varying covariates^9,10,27,29,32,34,35^. The factors which are known to affect relapse such as 6-TGN levels, IFX TLs, and timing of drug cessation are all variables that change with time after patient observation starts, therefore, the time-varying covariate approach is preferable for survival analysis in our study. This method increased the reliability of the study because it avoids biases associated with different timing of drug cessation in different patients.

The current study has a few limitations. First, this was a single-center, retrospective study and, consequently, had relatively unstructured follow-up of patients with certain limitations compared to studies using prospective design. However, all subjects received regular examinations such as endoscopy and biopsy on the same principles and IFX TLs or 6-TGN levels were examined at regular intervals. Therefore, the extraction of clinical disease activity using PCDAI, laboratory results, endoscopic and histologic results from medical records was possible at all outpatient visits. Although the decision to withdraw either IFX or AZA was not randomly assigned, there were no differences in disease activity and baseline as described in Table 2. Moreover, our findings are meaningful because these are real-world data from a single center cohort. Second, selection bias might have been introduced because the number of participating patients was relatively small and the observation period was not as long as might be desired. Also, because of adverse events such as lymphoma that may occur in male patients with long-term use of AZA, the group who withdrew AZA had a statistically larger number of male patients than the group who discontinued IFX, resulting in a selection bias of gender. However, in order to compensate for the shortcomings of the small number of subjects, it was attempted to increase the verification power through bootstrapping. Third, since multiple time-varying covariates made multivariate analysis too complicated to perform, only univariate results were presented.

Despite these limitations, our study reports the outcome of drug cessation in strictly selected cohort who sustained sufficient CR and achieved deep remission in real clinical settings. Maintaining the 6-TGN levels and IFX TLs high within the therapeutic range lowers the risk of clinical relapse and IFX cessation increase the risk of clinical relapse. In conclusion, even when applied in pediatric patients who meet strict criteria after a sufficient CR period and deep remission, IFX cessation in pediatric CD should be considered more carefully. However, withdrawal of AZA could be contemplated in selected pediatric patients with CD sustaining CR for at least two years and achieved deep remission.
